# Effects of FGFR gene polymorphisms on response and toxicity of cyclophosphamide-epirubicin-docetaxel-based chemotherapy in breast cancer patients

**DOI:** 10.1186/s12885-018-4951-z

**Published:** 2018-10-25

**Authors:** Lu Chen, Huijie Qi, Liudi Zhang, Haixia Li, Jie Shao, Haifei Chen, Mingkang Zhong, Xiaojin Shi, Ting Ye, Qunyi Li

**Affiliations:** 1 Department of Pharmacy, Huashan Hospital, Fudan University, Shanghai, China; 2 Department of Pathology, Huashan Hospital North, Fudan University, Shanghai, China; 3 Department of General Surgery, Huashan Hospital North, Fudan University, Shanghai, China; 4 Nursing Department, Huashan Hospital, Fudan University, Shanghai, China

**Keywords:** Fibroblast growth factor receptors, rs1966265, rs2981578, Single-nucleotide polymorphism, Neoadjuvant chemotherapy, Breast cancer

## Abstract

**Background:**

The chemotherapy resistance and toxicity of chemotherapy are major problems in breast cancer treatment. However, candidate biomarkers for predicting clinical outcomes and better prognosis remain lacking.

**Methods:**

In this study, we analyzed possible impact of 8 genetic variants of fibroblast growth factor receptor1–4 (FGFR1–4) on the treatment response and toxicities in 211 breast cancer patients. DNA was extracted from peripheral blood cells, and the genotypes were examined using the TaqMan Pre-Designed SNP Genotyping Assays.

**Results:**

The FGFR4 rs1966265 and FGFR2 rs2981578 contributed to clinical outcome of breast cancer treated with docetaxel–epirubicin-cyclophosphamide (CET)-based chemotherapy. For rs1966265, AA genotype had significant correlation with the clinical response to neoadjuvant chemotherapy (NCT) when compared with GG and AG/GG genotype (*P* = 0.019 and *P* = 0.004, respectively). Moreover, A allele of FGFR2 rs2981578 had significant rates of response (*P* = 0.025). In addition, rs2420946 CC genotype was associated with higher frequency of toxicities compared with TT and CT/TT genotypes (*P* = 0.038 and *P* = 0.019, respectively). Also, rs2981578 AG genotype showed higher frequency of toxicities compared with GG genotype (*P* < 0.0001).

**Conclusions:**

The results suggest these polymorphisms, especially rs1966265 and rs2981578, might be candidate pharmacogenomics factors to the response and prognosis prediction for individualized CET-based chemotherapy in breast cancer patients.

## Background

Breast cancer is a complex disease and the most common cancer in women worldwide with an estimated 1.7 million cases and 521,900 deaths in 2012 [1]. China, which has a massive population base, experienced the largest increase in incidence and mortality of breast cancer in recent years. Despite the recent progress in prevention, diagnosis, treatment of breast cancer and the consequent improvement in overall survival, the number of deaths increased 78% from 29,200 to 52,500 across entire age categories during 1990 to 2010 [2].

Presently, chemotherapy plays a crucial role in the systemic treatment of breast cancer. Yet, only ~ 13% patients benefit from chemotherapy in terms of prolonged overall survival, which implicates that individual genetic variation could be a significant factor for clinical responses [3]. Recently, numerous studies have demonstrated that single-nucleotide polymorphisms (SNPs) predict efficacy and toxicity of chemotherapy. Therefore, better predictive markers for improving personalized chemotherapy in breast cancer are urgently needed.

Fibroblast growth factor receptors (FGFRs) family, a sub family of receptor tyrosine kinases (RTKs), is comprised of four closely related genes (FGFR1–4) [4]. FGFR aberrations have been implicated in neoplastic human diseases and play important roles in tumor growth and progression [5]. FGFRs activating mutations and overexpression have been correlated with the development of various cancers, such as bladder, ovarian, breast, renal cell and squamous cell lung cancer [6]. The FGFRs are relevant to a broad spectrum of cellular processes in both embryonic and adult tissues. They induce diverse cellular responses beyond growth promoting signals in different target cells, such as human endothelial cells [7], pancreatic beta-cells [8], cancer cells [9–11] and vascular smooth muscle cells [12]. FGFR1 expression has been demonstrated to play imperative roles in mammary development and breast cancer tumourigenesis [13, 14]. Furthermore, amplification and overexpression of FGFR1 contributes to poor prognosis in luminal type breast cancers and serve as an independent predictor of poor outcome [15]. FGFR2 is a breast cancer susceptibility gene, and various variants of FGFR2 are significantly associated with the breast cancer risk [16]. Additionally, FGFR2 is essential in promoting breast tumorigenicity through self-renewal and maintenance of bipotent tumor-initiating cells [17]. FGFR3 could play an integral part in breast cancer development and be correlated with overall survival [18, 19]. FGFR4 is highly expressed in a subset of breast cancer cells and primary tumors, suggesting the pivotal role in cell survival via activation of PI3K/AKT [20].

Identification of single-nucleotide polymorphisms (SNPs) predicting toxicity and/or efficacy of chemotherapy might be of great clinical value [21]. Previous studies have investigated associations between genetic variants in FGFR families and cancer risk. FGFR1 amplification is a common genetic event and associated with tumor growth and survival in squamous cell lung cancer [22–24]. In addition, genetic variants of FGFR1 and FGFR2 may also be correlated not only with treatment toxicity but also with prognostic outcomes to cediranib therapy in nonsmall-cell lung cancer (NSCLC) [25]. Furthermore, the FGFR1 rs2288696 is the protective SNP with the highest statistical significance in ovarian cancer [26]. A present study has also investigated that FGFR1 rs2288696 may contribute to menarche timing in Ukrainian female, as well as early menarche onset is a known risk factor for breast and ovarian cancer in late adulthood [27]. As others have already shown, an intronic single nucleotide polymorphism in FGFR2 rs2981582 was identified as being associated with breast cancer risk. The risk appears to be consistent in either Han Chinese population or Caucasian, Mexican and Hispanic women [16]. Many studies have demonstrated that FGFR3 gene mutations were associated with many epithelial cancers, including colorectal cancer, bladder cancer, cervical carcinoma and nasopharyngeal carcinoma [28]. Additionally, a prospective Spanish study has identified the prevalence and prognostic significance of FGFR3 gene mutations in 772 superficial urothelial tumors. Furthermore, multivariate analysis of all the superficial tumors did establish that FGFR3 mutations were associated with an increased risk of recurrence [28]. A meta and pooled analyses provide evidence of a role for the FGFR4 Gly388Arg polymorphism in modulating patients’ outcome in different types of cancer, thus offering to clinicians a new marker to predict predisposition to poor survival in cancer patients [29]. Moreover, the FGFR4 Arg388 allele may be closely interrelated with incidence of febrile neutropenia during neoadjuvant CET chemotherapy and is possibly useful as a patient related risk factor for febrile neutropenia [30].

Despite the well-known association between FGFR polymorphisms and the risk of breast cancer, few genetic variants of FGFR SNPs had been found to be associated with clinical outcomes in CET-based chemotherapy of breast cancer. To fill this gap, we selected eight SNPs of FGFRs which have been previously described to be associated with breast cancer risk [31], and evaluated the possible correlations between the SNPs and the efficacy/ toxicity of CET-based chemotherapy in patients with Chinese breast cancer.

## Methods

### Study population and chemotherapy

A total of 211 women with histologically proven invasive breast carcinoma were recruited to this study. These patients underwent surgery in the Department of Breast Disease at Shanghai Huashan Hospital (Shanghai, China) between September 2015 and November 2016. Every patient received CET-based chemotherapy. Of all the patients, 120 received adjuvant chemotherapy (ACT) and 91 received neoadjuvant chemotherapy (NCT). Patients received CET (cyclophosphamide 600 mg/m^2^ i.v. in 1 h, epirubicin 75 mg/m^2^ i.v. in 15 min and docetaxel 75 mg/m^2^ i.v. in 1 h) on the first day of each of four 21-day cycles chemotherapy. All patients filled the informed consent forms and agreed to have their samples used for research purposes. The procedures of this study were approved by the Huashan Hospital Institutional Review Board and the approved number of ethic committee was 2017–017.

### Response and toxicity evaluation criteria

The effect of FGFR gene polymorphisms on the response was evaluated in patients (90) receiving only CET-based NCT, whereas that on toxicities was evaluated in patients (211) receiving both CET-based NCT (90) and ACT (120). Tumor response to chemotherapy was assessed after the first two or three cycles NCT and determined by the Response Evaluation Criteria in Solid Tumors (RECIST) criteria [32]. For data analysis, patients were categorized into two groups as responders (complete + partial response) and non-responders (stable + progressive disease). Toxicities were graded according to the National Cancer Common Terminology Criteria for Adverse Events (CTCAE v4.0) [33]. TNM stages were determined according to the 8th American Joint Committee on Cancer (AJCC) TNM system.

### DNA samples

A peripheral blood sample from each patient was collected. Peripheral blood samples were centrifuged at 3000 rpm/min for 10 mins and then the blood serum was separated out. Genomic DNA was extracted after red cell lysis and isolated from peripheral blood leukocytes using erythrocyte lysate of TIAamp® Blood DNA Kit (Tiangen, Beijing, China).

### Clinicopathological factors

The expression of ER and PR was detected using immunohistochemistry methods and scored according to the Allred system [34]. HER2 positivity was defined as 3 + staining in immunohistochemistry or unequivocal HER2 gene amplification by fluorescent in situ hybridization (FISH). Immunohistochemistry for P63 expression was realised to be negative if positive staining was detected in less than 5% of all the cells [35]. The optimal cut-off to classified the status of Ki67 was defined as 20% (i.e. ≥ 20% staining was defined as Ki67hi), according to the 2015 recommendations [36].

### SNP selection and genotyping

We selected 8 SNPs of the FGFR1–4 genes according to the following criteria: 1) the minor allele frequencies (MAF) of these SNPs were greater than 10%, 2) SNPs have been associated with breast cancer risk or clinical outcome in prior studies. The details of the genetic variants studies, minor allele frequency (MAF) and primer sequences are summarized in Table 1. Eight single nucleotide polymorphisms was determined using the TaqMan Pre-Designed SNP Genotyping Assays (Applied Biosystems, Foster City, CA) following manufacturer’s instructions. Polymerase chain reaction (PCR) was performed in a final volume of 5-μl containing TaqMan Universal PCR Master Mix, TaqMan SNP Genotyping Assays Mix, and 20 ng of genomic DNA. PCR products were sequenced using an ABI Prism® 3730 DNA Analyzer (Applied Biosystems, Foster City, CA) and data were collected using GeneMapper® version 4.0 software (Applied Biosystems, Foster City, CA).

**Table 1:** Information for the SNPs genotyped in this study

Gene	Ref SNP ID	Alleles	MAF	Primer sequences
FGFR1	rs2288696	C/T	0.14	5’-GGTGCCCTATCTGCTCTG-3′, 5’-CTTGGTGGGATGGAAACT-3’
FGFR2	rs2981582	C/T	0.40	5’-TCTGTCATCAGTAGGGAATA-3′, 5’-ATCACCAAGAGGCAGTTC-3’
	rs1219648	A/G	0.41	5’-ACATCCCTTGTTCTCGTT-3′, 5’-ATTGCCTTGGCTATTCAG-3’
	rs2420946	T/C	0.44	5’-TTTGGTGGAAGAGTCAGA-3′, 5’-GTCCCAGAGGATTTGTTT-3’
	rs2981579	T/C	0.49	5’-TGTGACTCCCTTCATCGT-3′, 5’-TGGTCTATTTCTCAATCCCTA-3’
	rs2981578	G/A	0.37	5’-CTGCTTTGGAGGATTGTGA-3′, 5’-GAGACTGGGAAACAGATGGT-3’
FGFR3	rs743682	G/A	0.17	5’-TACAAAGACCTCGTGAAATGG-3′, 5’-CAAATCCAGACTCGCTTCC-3’
FGFR4	rs1966265	G/A	0.23	5’-GCCGCCTTGTCAGTTGTG-3′, 5’-TCCTGGTGCCCACTCTTCC-3’

*SNP* single nucleotide polymorphism, *MAF* minor allele frequency

### Statistical analysis

The primary end point of the study was to analyze the association between the genetic polymorphisms and clinical outcome and toxicity. Chi-square (χ2) test for categorical variables was used to estimate population distribution characteristics and compare the differences in the genotype frequencies between patients with different treatment outcomes and toxicity. Meanwhile, chi-square (χ2) test was also used to analyze associations between an individual SNP and clinicopathological parametrs. Odd ratios (ORs) with 95% confidence intervals (95%CIs) were calculated by unconditional logistic regression to analyze the associations between genetic polymorphisms and clinical outcome and toxicity. ORs with 95%CIs were adjusted for confounding variables like age, menstrual status, clinical TNM stage, histology, tumor grade, HER2 status and hormone receptor (HR). *P*-value < 0.05 was considered to be statistically significant, while *P* < 0.100 was treated as an indicator for trend in given analysis. All statistical analyses were conducted using the SPSS version 16.0 for Windows (SPSS, Chicago, IL, USA). The Hardy–Weinberg equilibrium (HWE) was examined in patients (211) receiving both CET-based NCT (90) and ACT (120) by using the chi-squared test.

## Results

### Patient characteristics and clinical predictors

The discovery cohort included 211 patients who received 4 cycles of anthracycline-based CET regimen. The demographic and clinical characteristics of patients in study were listed in Table 2. The clinicopathological characteristics and the association with chemotherapy treatment response and toxicity are presented in Table 3. According to the RECIST criteria, response assessment was made in 90 patients who were given NCT, and it was observed that 29 (32%) patients were responders and 61 (68%) patients were non-responders. Then, toxicity was assessed in 211 patients who were given NCT and ACT according to the CTCAE. Among them, 112 (48%) patients suffered from grade 3–4 toxicity while 109 (52%) patients had no grade 3–4 toxicity. As shown in Table 3, patients who responded to NCT were comparable to those who did not respond in terms of age, menstrual status, tumor status, lymph node status, tumor grade, HR status, triple-negative breast cancer (TNBC), P63, Ki67, and Her-2/neu expression (*P* > 0.05). Similarly, patients who suffered from grade 3–4 toxicity did not differ significantly with those no or grade 1–2 toxicity in terms of age, menstrual status, lymph node status, tumor grade, HR status, TNBC, P63, Ki67, and Her-2/neu expression (*P* > 0.05). However, patients who suffered from grade 3–4 toxicity varied significantly in terms of tumor status (Table 3, *P* < 0.001).

**Table 2:** Patient characteristics (*N* = 211)

	Mean ± SD	N (%)
Age at diagnosis (years)	54.278 ± 10.163	
Menopausal state		
premenopausal		63 (29.9)
postmenopausal		148 (70.1)
Age at postmenopause (years)	52.367 ± 8.132	
Height (cm)	159.672 ± 5.369	
Weight (kg)	62.483 ± 8.859	
BMI (kg.m^−2^)	24.114 ± 3.156	
Parity		194 (91.9)

*BMI* body mass index

**Table 3:** The clinic-pathological parameters among patients with different treatment response and toxicity

Characteristic	Response to chemotherapy(*N* = 90)		Toxicity to chemotherapy(*N* = 211)			
	Response (*N* = 29)	Non-response (*N* = 61)	*p*	Toxicity (*N* = 102)	No toxicity (*N* = 109)	*P*
Age in years(mean ± SD)			0.539			0.195
≤ 45	4(13.8)	10(16.4)		17(16.7)	22(20.2)	
46–55	10(34.5)	27(44.3)		39(38.2)	29(26.6)	
> 55	15(51.7)	24(39.3)		46(45.1)	58(53.2)	
Menstrual status			0.516			0.891
Premenopausal	8(27.6)	21(34.4)		30(29.4)	33(30.3)	
Postmenopausal	21(72.4)	40(65.6)		72(70.6)	76(69.7)	
T-status,n(%)			0.250			<0.001*
T1	/	/		/	/	
T2	1(3.4)	5(8.2)		17(16.7)	74(67.9)	
T3	27(93.1)	56(91.8)		84(82.4)	32(29.4)	
T4	1(3.4)	0(0)		1(1.0)	3(2.8)	
N-status,n(%)			0.209			0.168
N0	7(24.1)	27(44.3)		38(37.3)	27(24.8)	
N1	13(44.8)	23(37.7)		40(39.2)	58(53.2)	
N2	6(20.7)	9(14.8)		18(17.6)	19(17.4)	
N3	3(10.3)	2(3.3)		6(5.9)	5(4.6)	
Tumor grade			0.066			0.078
Grade I	/	/		/	/	
Grade II	7(24.1)	27(44.3)		41(40.2)	57(52.3)	
Grade III	22(75.9)	34(55.7)		61(59.8)	52(47.7)	
ER status			0.050			0.650
Positive	24(82.8)	38(62.3)		72(70.6)	80(73.4)	
Negative	5(17.2)	23(37.7)		30(29.4)	29(26.6)	
PR status			0.476			0.995
Positive	24(82.8)	38(62.3)		59(57.8)	63(57.8)	
Negative	5(17.2)	23(37.7)		43(42.2)	46(42.2)	
Her2 status			0.883			0.525
Positive	10(34.5)	22(36.1)		35(34.3)	42(38.5)	
Negative	19(65.5)	39(63.9)		67(65.7)	67(61.5)	
TNBC			0.618			0.428
yes	2(6.9)	15(24.6)		16(15.7)	13(11.9)	
no	27(93.1)	46(75.4)		86(84.3)	96(88.1)	
P63			0.967			0.281
positive	1(3.4)	2(3.3)		3(2.9)	1(0.9)	
negative	28(96.6)	59(96.7)				
Ki-67			0.324	17(16.7)	22(20.2)	0.195
< 20%	7(24.1)	21(34.4)		39(38.2)	29(26.6)	
≥ 20%	22(75.9)	40(65.6)		46(45.1)	58(53.2)	

*T* tumor stage, *N* nodes stage, *TNBC* triple negative breast cancer, *Her 2* human epidermal growth factor receptor 2; * A *P* value < 0.05 was considered ststistically significant

### Associations between SNPs and clinical outcomes

Considering the potential of FGFR gene polymorphisms to predict therapy response, we distributed the relationship between 8 selected SNPs of FGFR and the efficacy of chemotherapy in Table 4. The results of unconditional logistic regression analysis revealed that the genotype distribution of the FGFR4 rs1966265 polymorphisms was significantly different between the responders and non-responders. For rs1966265, AA genotype had significant correlation with the clinical response to NCT when compared with GG genotype (adjusted OR = 0.17, 95% CI = 0.03–0.74, *P* = 0.019). Furthermore, a significant correlation was observed in the recessive model (AA vs. AG/GG: adjusted OR = 1.33, 95% CI = 1.88–25.19, *P* = 0.004). Moreover, the individual carrying A allele had significantly rates of response to NCT when compared to those carrying G allele (A vs. G: adjusted OR = 0.43, 95%CI = 0.23–0.81, *P* = 0.009).

**Table 4:** Univariate analyses of the associations between FGFR polymorphisms and the response of neoadjuvant chemotherapy

Target Gene	Polymorphisms/Genotype	Nonresponders *N* = 29(32%)	Responders *N* = 61(68%)	OR (95% CI)	*P*	*P*_*HWE*_
FGFR1	rs2288696					0.014
	C/C	25(86.2)	50(82.0)	Reference	1.000	
	C/T	1(3.4)	11(18.0)	1.00 (0.28–3.64)	0.999	
	T/T	3(10.3)	0(0)	0	0.615	
	Dominat model	4(13.8)	4(6.6)	0.73(0.21–2.52)		
	Recessive model	26(89.7)	61(100)	0	1.000	
	Alleles					
	C	51(87.9)	111(91.0)	Reference	0.344	
	T	7(12.1)	11(9.0)	0.57(0.18–1.82)	0.999	
FGFR2	rs2981582					0.343
	C/C	11(37.9)	31(50.8)	Reference		
	C/T	15(51.7)	21(34.4)	2.01(0.78–5.23)	0.151	
	T/T	3(10.3)	9(14.8)	0.94(0.22–4.11)	0.934	
	Dominat model	18(62.1)	30(49.2)	1.69(0.69–4.17)	0.254	
	Recessive model	26(89.7)	52(85.2)	1.50(0.37–6. 02)	0.567	
	Alleles					
	C	37(63.8)	83(68.0)	Reference		
	T	21(36.2)	39(32.0)	1.21(0.63–2.33)	0.573	
	rs1219648					0.700
	A/A	9(31.0)	19(31.1)	Reference		
	A/G	16(55.2)	30(49.2)	1.13(0.42–3.06)	0.816	
	G/G	4(13.8)	12(19.7)	0.70(0.18–2.80)	0.618	
	Dominat model	20(69.0)	42(68.9)	1.01(0.39–2.61)	0.99	
	Recessive model	25(86.2)	49(80.3)	1.53(0.45–5.24)	0.498	
	Alleles					
	A	34(58.6)	68(55.7)	Reference		
	G	24(41.4)	54(44.3)	0.89(0.47–1.67)	0.715	
	rs2420946					0.377
	T/T	1(3.4)	11(18.0)	Reference		
	C/T	18(62.1)	29(47.5)	6.83(0.81–57.45)	0.077	
	C/C	10(34.5)	21(34.4)	5.24(0.59–46.40)	0.137	
	Dominat model	28(96.6)	50(82.0)	6.16(0.76–50.24)	0.090	
	Recessive model	22(75.9)	54(88.5)	1.00 (0.39–2.53)	0.996	
	Alleles					
	T	20(34.5)	51(41.8)	Reference		
	C	38(65.5)	71(58.2)	1.37(0.71–2.61)	0.348	
	rs2981579					0.060
	T/T	7(24.1)	18(29.5)	Reference		
	C/T	12(41.4)	24(39.3)	1.29(0.42–3.92)	0.659	
	C/C	10(34.5)	19(31.1)	1.35(0.42–4.32)	0.610	
	Dominat model	22(75.9)	43(70.5)	2.39(0.96–5.99)	0.062	
	Recessive model	26(89.7)	51()	0.86(0.34–2.20)	0.752	
	Alleles					
	T	26(44.8)	60(49.2)	Reference		
	C	32(55.2)	62(50.8)	1.19(0.64–2.23)	0.585	
	rs2981578					0.060
	G/G	10(34.5)	34(55.7)	Reference		
	A/G	12(41.4)	20(32.8)	2.04(0.75–5.57)	0.164	
	A/A	7(24.1)	7(11.5)	3.40(0.96–12.02)	0.058	
	Dominat model	19(65.5)	27(44.3)	2.39(0.96–5.99)	0.062	
	Recessive model	22(75.9)	54(88.5)	0.41(0.13–1.30)	0.129	
	Alleles					
	G	32(55.2)	88(72.1)	Reference		
	A	26(44.8)	34(27.9)	2.10(1.10–4.03)	0.025*	
FGFR3	rs743682					0.198
	G/G	25(86.2)	49(80.3)	Reference		
	A/G	3(10.3)	11(18.0)	0.54(0.14–2.09)	0.368	
	A/A	1(3.4)	1(1.6)	1.96(0.12–32.67)	0.639	
	Dominat model	4(13.8)	12(19.7)	0.65(0.65–0.19)	0.498	
	Recessive model	27(93.1)	61(100)	0.47(0.03–7.74)	0.595	
	Alleles					
	G	53(91.4)	109(89.3)	Reference		
	A	5(8.6)	13(10.7)	0.79(0.27–2.34)	0.671	
FGFR4	rs1966265					0.343
	G/G	8(27.6)	12(19.7)	Reference		
	A/G	18(62.1)	22(36.1)	1.23(0.41–3.65)	0.713	
	A/A	3(10.3)	27(44.3)	0.17(0.04–0.74)	0.019*	
	Dominat model	21(72.4)	49(80.3)	1.16(0.64–1.80)	0.401	
	Recessive model	26(89.7)	34(55.7)	6.88(1.88–25.2)	0.004*	
	Alleles					
	G	34(58.6)	46(37.7)	Reference		
	A	24(41.4)	76(62.3)	0.43(0.23–0.81)	0.009*	

*OR* odds ratio, *95% CI* 95% confidence interval, * A *P* value < 0.05 was considered ststistically significant

For FGFR2 rs2981578, the individual carrying A allele had significant rates of response when compared with those carrying G allele (A vs. G: adjusted OR = 2.10, 95% CI = 1.10–4.03, *P* = 0.025). However, no significant associations were observed between the other six SNPs examined and the response to neoadjuvant CET chemotherapy (Table 4).

### Correlation between FGFR SNP s and adverse events

Having confirming that FGFR SNPs (rs1966265 and rs2981578) were associated with clinical response, we further investigated the potential association between FGFR SNPs and clinical toxicities. As shown in Table 5, toxicities of 211 studied patients according to the NCI-CTCAE version 4.0 are summarized. Overall, grade 3–4 toxicities were observed in 102 patients (48%). Grade 3–4 leucopenia developed in 95 (93.1%) patients with 90 (88.2%) neutropenia, as grade 3–4 vomiting did in 18 patients (17.4%). No treatment related mortality was encountered in the study.

**Table 5:** drug toxicity and adverse events

Adverse events	No toxicity (grade0–2) (*N* = 109)	Toxicity(grade 3–4) (*N* = 102)
Hematological		
Leucopenia	98	95
Neutropenia	93	90
Anemia	8	1
Thrombocytopenia	2	1
Gastrointestinal		
Nausea	47	0
Vomiting	86	18
Diarrhea	16	13
Skin rash	6	2
hepatotoxicity	3	0
Renal toxicity	1	0

Next, we analyzed the association between toxicity outcomes and FGFR SNPs (Table 6). The results of unconditional logistic regression analysis demonstrated that CC genotype of FGFR1 rs2420946 was associated with higher frequency of toxicities compared with TT genotype (adjusted OR = 2.35, 95%CI = 1.05–5.28, *P* = 0.038). The relevance of rs2420946 with total toxicity in recessive model was (CC vs. CT/TT: adjusted OR = 0.51, 95%CI = 0.29–0.90, *P* = 0.019). All the results show that individuals with the C allele of FGFR1 rs2420946 presented a greater risk of overall toxicity for CET chemotherapy in breast cancer (adjusted OR = 1.65, 95% CI = 1.11–2.46, *P* = 0.013). Meanwhile, for FGFR2 rs2981578, AG genotype was associated with higher frequency of toxicities compared with GG genotype (adjusted OR = 4.91, 95% CI = 2.65–9.08, *P* < 0.0001). The correlation of rs2981578 with total toxicity in dominant model was (GG vs. AG/AA: adjusted OR = 2.57, 95% CI = 0.99–6.69, *P* < 0.0001). All the results show that individuals with the A allele of FGFR2 rs2981578 presented a greater risk of overall toxicity for CET chemotherapy in breast cancer (adjusted OR = 2.37, 95% CI = 1.55—3.61, *P* < 0.0001).

**Table 6:** Genotypes of 8 SNPs of FGFR gene and their associations with adverse events

Target Gene	Polymorphisms/Genotype	Toxicity(grade 3–4) *N* = 102(48%)	No toxicity (grade1–2) *N* = 109(52%)	OR (95%CI)	*P*	*P*_*HWE*_
FGFR1	rs2288696					0.035
	C/C	85(83.3%)	88(80.7%)	Reference		
	C/T	14(13.7%)	19(17.4%)	0.76(0.36—1.62)	0.480	
	T/T	3(2.9%)	2(1.8%)	1.55(0.25—9.53)	0.634	
	Dominat model	17(16.7%)	21(19.3%)	0.84(0.41—1.70)	0.624	
	Recessive model	99(97.1%)	107(98.2%)	0.62(0.10—3.77)	0.601	
	Alleles					
	C	184(90.2%)	195(89.4%)	Reference		
	T	20(9.8%)	23(10.6%)	0.92(0.49—1.73)	0.800	
FGFR2	rs2981582					0.138
	C/C	52(51.0%)	47(43.1%)	Reference		
	C/T	37(36.3%)	47(43.1%)	0.71(0.40—1.28)	0.253	
	T/T	13(12.7%)	15(13.8%)	0.78(0.34—1.82)	0.569	
	Dominat model	50(49.0%)	62(56.9%)	0.73(0.42—1.25)	0.253	
	Recessive model	89(87.3%)	94(86.2%)	1.09(0.49—2.43)	0.828	
	Alleles					
	C	141(69.1%)	141(64.7%)	Reference		
	T	63(30.9%)	77(35.3%)	0.82(0.55—1.30)	0.333	
	rs1219648					0.726
	A/A	34(33.3%)	33(30.3%)	Reference		
	A/G	49(48.0%)	57(52.3%)	0.83(0.45—1.54)	0.562	
	G/G	19(18.6%)	19(17.4%)	0.97(0.44—2.15)	0.941	
	Dominat model	68(66.7%)	76(69.7%)	0.87(0.49—1.55)	0.634	
	Recessive model	83(81.4%)	90(82.6%)	0.92(0.46—1.86)	0.821	
	Alleles					
	A	117(57.4%)	123(56.4%)	Reference		
	G	87(42.6%)	95(43.6%)	0.96(0.66—1.42)	0.847	
	rs2420946					0.172
	T/T	13(12.7%)	22(20.2%)	Reference		
	C/T	39(38.2%)	51(46.8%)	1.29(0.58—2.89)	0.529	
	C/C	50(49.0%)	36(33.0%)	2.35(1.05—5.28)	0.038*	
	Dominat model	89(87.3%)	87(79.8%)	1.73(0.82—3.65)	0.150	
	Recessive model	52(51.0%)	73(67.0%)	0.51(0.29—0.90)	0.019*	
	Alleles					
	T	65(31.9%)	95(43.6%)	Reference		
	C	139(68.1%)	123(56.4%)	1.65(1.11—2.46)	0.013*	
	rs2981579					0.196
	T/T	29(28.4%)	25(22.9%)	Reference		
	C/T	38(37.3%)	58(53.2%)	0.57(0.29—1.11)	0.096	
	C/C	35(34.3%)	26(23.9%)	1.16(0.56—2.43)	0.692	
	Dominant model	73(71.6%)	84(77.1%)	0.75(0.40—1.39)	0.361	
	Recessive model	67(65.7%)	83(76.1%)	0.60(0.33—1.09)	0.095	
	Alleles					
	T	96(47.1%)	108(49.5%)	Reference		
	C	108(52.9%)	110(50.5%)	1.11(0.75—1.62)	0.610	
	rs2981578					0.909
	G/G	30(29.4%)	70(64.2%)	Reference		
	A/G	61(59.8%)	29(26.6)	4.91(2.65—9.08)	< 0.0001*	
	A/A	11(10.8%)	10(9.1%)	2.57(0.99—6.69)	0.054	
	Dominant model	72(70.6%)	39(35.8%)	2.57(0.99–6.69)	< 0.0001*	
	Recessive model	91(89.2%)	99(90.8%)	0.84(0.34–2.06)	0.697	
	Alleles					
	G	121(59.3%)	169(82.8%)	Reference		
	A	83(40.7%)	49(24.0%)	2.37(1.55—3.61)	< 0.0001*	
FGFR3	rs743682					0.682
	G/G	85(83.3%)	92(84.4%)	Reference		
	A/G	15(14.7%)	17(15.6%)	0.96(0.45—2.03)	0.955	
	A/A	2(2.0%)	0	0.00	0.999	
	Dominant model	17(16.7%)	17(15.6%)	1.08(0.52—2.26)	0.833	
	Recessive model	100(98.0%)	109(100.0%)	0.00	0.999	
	Alleles					
	G	185(90.7%)	201(92.2%)	Reference		
	A	19(9.3%)	17(7.8%)	1.21(0.61—2.41)	0.578	
FGFR4	rs1966265					0.998
	G/G	22(21.6%)	19(17.4%)	Reference		
	A/G	46(45.1%)	58(53.2%)	0.69(0.33—1.42)	0.307	
	A/A	34(33.3%)	32(29.4%)	0.92(0.42—2.00)	0.829	
	Dominant model	80(78.4%)	90(82.6%)	0.77(0.39—1.52)	0.449	
	Recessive model	68(66.7%)	77(70.6%)	0.83(0.46—1.49)	0.534	
	Alleles					
	G	90(44.1%)	96(44. %)	Reference		
	A	114(55.9%)	122(56.0%)	1.00(0.68—1.46)	0.987	

*OR* odds ratio, *95% CI* 95% confidence interval, * A *P* value < 0.05 was considered ststistically significant

### Association between rs1966265 and clinicopathologic variables

Based on the striking observation that rs1966265 might serve as an independent predictive factor for clinical response to NCT with CET regimen, we performed an exploratory analysis to investigate the correlation between rs1966265 and clinicopathologic variables. With respect to other important clinicopathological factors in breast cancer such as grading, HR status, Her2/neu status, and age, however, no statistically significant correlations were observed. The associations between FGFR4 genotype and clinicopathological prognostic factors are summarized in Table 7.

**Table 7:** Clinicopathological parameters analysis according to rs1966265 genetic polymorphism in breast cancer patients

Clinicopathological parameters	rs1966265(N = 90)				
	GG	AG &AA	OR	95%CI	*P*
T-status,n(%)					
≤ T2	3(3.3)	3(3.3)			
> T2	17(18.9)	67(74.4)	3.941	0.730–21.284	0.111
N-status,n(%)					
N0+ N1	15(6.7)	55(61.1)			
N2+ N3	5(5.6)	15(16.7)	0.818	0.256–2.615	0.735
Tumor grade					
GradeII	8(8.9)	26(28.9)			
GradeIII	12(13.3)	44(48.9)	1.128	0.408–3.121	0.816
ER					
positive	16(17.8)	46(51.1)			
negative	4(4.4)	24(26.7)	2.087	0.628–6.941	0.230
PR					
positive	13(14.4)	38(42.2)			
negative	7(7.8)	32(35.6)	1.564	0.557–4.390	0.396
Her2-neu stastus					
positive	7(7.8)	25(27.8)			
negative	13(14.4)	45(50.0)	0.969	0.342–2.744	0.953
TNBC					
Yes	3(3.3)	13(14.4)			
No	17(18.9)	57(63.3)	1.292	0.328–5.072	0.713
P63					
positive	2(2.2)	1(1.1)			
negative	18(20.0)	69(76.7)	7.667	0.658–89.367	0.104
Ki-67					
positive	3(3.3)	25(27.8)			
negative	17(18.9)	45(50.0)	0.318	0.085–1.190	0.089

*T* tumor stage, *N* nodes stage, *TNBC* triple negative breast cancer, *Her 2* human epidermal growth factor receptor 2

## Discussion

The principal finding of this trial is that two SNPs (rs1966265 and rs2981578) contribute to clinical outcome of breast cancer treated with CET-based chemotherapy. In addition, our results showed for the first time that rs2420946 CC genotype and rs2981578 GG genotype demonstrated the association with chemotherapy toxicities. Given the critical role of FGFR genetic variations in breast cancer, these polymorphisms, especially rs1966265 and rs2981578, might be vital response and prognostic indicators for breast cancer.

It is generally known that the development and progression of breast cancer is a complicated process that involves both epigenetic and genetic factors [37]. Randomized trials [38] have proved the neoadjuvant chemotherapy (NCT) is the standard of care for patients with inflammatory and inoperable breast cancer, which has been a widely accepted modality for treatment of early breast cancer. In addition to decreasing relapse and mortality of tumor, neoadjuvant chemotherapy is also effective with respect to enhancing disease-free and overall survival [38, 39]. Furthermore, for the reason that NCT offers the unique opportunity to directly measure a tumour’s response to therapy, it is one of the best way to evaluate new predictive and prognostic factors in breast cancer [40]. Practice has proven that anthracyclines doxorubicin (DOXO) or epirubicin (EPI) and taxanes (T) are among the most effective cytotoxic treatments developed for the comprehensive treatment of breast cancer. Meanwhile, variability in quality-of-life impairing toxicity or life-threaten side effects remain a major problem for patients with neoadjuvant chemotherapy. With some patients’ poor response to chemotherapy, suspectable to toxicity of chemotherapy, better predictive markers for NCT are urgently needed.

SNPs in genes involving pharmacokinetics such as drug metabolism and drug efflux transporters can modulate the metabolism and distribution of drugs and therefore influence response to chemotherapy. Nowadays, a growing number of research has indicated that other genes involving the occurrence and development of tumor are also connected with chemotherapy outcomes and toxicities. More recently, accumulating evidence indicates that FGFs and FGFRs may promote cancer progression by inducing mitogenic and survival signals, as well as epithelial-mesenchymal transition invasion and tumor angiogenesis [41, 42]. FGFR alterations are detected in a variety of human cancers, such as prostate, bladder, breast, lung and endometrial cancers. Furthermore, the correlations of FGFR family genetic variations with the susceptibility and clinicopathological features of cancer patients have been intensely investigated. Nevertheless, little is known about the associations between FGFR SNPs and the clinical outcomes of breast cancer patients after CET regimen.

Thereafter, the present study firstly evaluated the effect of eight selected potentially functional SNPs of FGFR on chemotherapy efficacy of CET in breast cancer patients. The major finding was a significant association of FGFR4 rs1966265 AA genotype and A allele and FGFR2 rs2981578 A alleles with an increased chemosensitivity in NCT of breast cancer patients. The Chi-square test has showed no deviation from Hardy Weinberg equilibrium for the two SNPs in patients (211) receiving both CET-based NCT (90) and ACT (120), suggesting that the association does not result from population admixture or genotyping errors.

Our finding that FGFR4 rs1966265 polymorphism predicted the individual clinical response to the CET regimen is in accordance with the results from a previous study [42]. In the susceptibility analysis of breast cancer, for rs1966265, Jiang et al. demonstrated that the AG and GG genotypes conferred a significantly increased risk for breast cancer compared to the AA genotype in the dominant model (OR = 1.66, 95%CI = 1.31–2.11, *P* < 0.001) [42]. Several other studies have also investigated the possible relationship between the rs1966265 polymorphism and disease risk. Chen et al. [43] demonstrated that FGFR4 rs1966265 was associated with the progression of cervical normal tissues to precancerous lesions in Asian women. Rezvani et al. [44] showed rs1966265 in FGFR4 is a possible genetic key variant in respiratory distress syndrome (*P* = 0.003) and colonic transit (*P* = 0.043), respectively [45]. Camilleri et al. revealed that FGFR4 rs1966265 was associated with stool level of bile acid (BA) (*P* = 0.032) [46]. Taken together, our results presented here suggested that FGFR4 rs1966265 polymorphisms could be employed as a biomarker for the prediction of clinical outcomes of CET-based chemotherapy in breast cancer patients.

Nevertheless, it is worthy to note that rs1966265 (Val 10 Ile) is a missense variant in FGFR4. In another investigation focused on FGFR4 missense variant, the GG of FGFR4 rs351855 (Gly 388 Arg) showed significantly better overall survival than the AG or AA among 273 colon cancer patients, regardless of adjuvant treatment [47]. Specially, overexpression of Arg388 significantly increased cell proliferation and changes in epithelial to mesenchymal transition markers compared with cells overexpressing the Gly388 allele in transfected colon cancer cells [47]. Although the amino acid change was estimated to be benign by PolyPhen (http://genetics.bwh.harvard.edu/pph2), the effects of the FGFR4 Val10 and Ile10 alleles on cancer cell biology such as proliferation and migration should also be examined in further investigation [48].

Recently, lots of studies have reported the association between rs2981578, rs2981579 and breast cancer risk in any genetic model [49]. For rs2981578, Chen et al. [37] revealed that rs2981578 are associated with a decreased risk of breast cancer, which is contrary to the previous reports [35, 50], indicating that the difference of the associations is due to different minor alleles among different ethnicities. Also, there are interactions between rs2981578 and the age, BMI, and family history of breast cancer. In the present study, rs2981578 A allele predicted the individual chemosensitivity to CET-based regimen of breast cancer patients compared with G allele, manifesting it as a potential prognostic indicator in NCT efficacy of breast cancer.

Generally, the anthracyclines and taxanes are associated with some life-threatening side effects. The most worrisome toxicities are myelosuppression and cardiac toxicity [39]. Myelosuppression was usually measured in the form of neutropenia [51], it is noteworthy that patients with neutropenia are at risk of developing life-threatening infections [43]. Patients experiencing febrile neutropenia during chemotherapy will often give rise to dose reductions or delays [44], and thus receive a potentially less effective treatment [52]. Also, cardiac toxicity limits the duration of drug treatment, particularly at high cumulative doses [53]. As a result, it is of great value to seek better predictive markers for identifying patients who are inclined to suffer from severe toxicity caused by chemotherapy.

Notably, our study revealed that the CC genotype of FGFR1 rs2420946 significantly increased the risk of CET-induced leucopenia, neutropenia and gastrointestinal toxicity in comparison to TT genotype. Meanwhile, FGFR2 rs1981578 was also associated with higher frequency of toxicities in dominant model. Thus far, only one previous study monitored the effect TT genotype of FGFR4 rs351855 had a tendency toward higher incidence of febrile neutropenia during NCT. Charehbili et al. genotyped 187 DNA samples extracted from patients with stage II/III HER2-negative breast cancer. Of note, the study showed that patients with a TT genotype in rs351855 (FGFR4) may be at a tendency toward higher incidence of febrile neutropenia during neoadjuvant TAC chemotherapy [30]. It is our hope that this study will add the body of literature for prediction the FGFRs genetic polymorphisms lead to individual toxicities in response to CET-based regimen.

CET-containing agents exert cytotoxic effects through the creation of cyclophosphamide-DNA crosslinks and the induction of cell cycle arrest and ultimately apoptosis if not properly repaired. Ghayad et al. [54] showed that the PI3K/PTEN/AKT pathway has been linked to promotion of survival in breast cancer cells, resistance to chemotherapy, resistance to endocrine therapy, and has been associated with poor prognosis. Furthermore, Starska et al. [55] revealed that PI3K/PTEN/AKT is FGFs and their receptors’ downstream regulator signaling pathway. Recently, genetic variations within the PI3K/PTEN/AKT have been reported to modulate clinical outcomes in esophageal cancer and lung cancer patients [56, 57]. Thus, genetic variations in the genes encoding these important molecules may modulate signaling through this pathway and result in variation in the development of toxicity or clinical outcomes following CET-based therapy. To conclude, further investigation is needed to confirm the predictive values of FGFR polymorphism in PI3K/PTEN/AKT signaling pathway.

Due to the limited samples in the present trial, a well-designed study with a larger number of BC patients is needed to verify the predictive values of FGFR SNPs. To address this we are continuing to collect specimens from breast cancer patients to further validate our results as well as extending our studies to an independent patient cohort.

## Conclusion

This study firstly provides evidence of significant association between FGFR genetic variants and the responsiveness of the breast cancer patients to CET chemotherapy. These data suggest 4 FGFR polymorphisms had a main effect on clinical responses and toxicities, and may be employed as candidate biomarkers for predicting clinical outcomes and achieving better prognosis in BC patients with CET regimen.

## Abbreviations

ACT: adjuvant chemotherapy
AKT: protein kinase B
BMI: body mass index
CET: docetaxel, epirubicin and cyclophosphamide
CTCAE: Common Terminology Criteria for Adverse Events
FGFR: Fibroblast growth factor receptor
i.v.: intravenous injection
mTOR: the mammalian target of rapamycin
NACT: neoadjuvant adjuvant chemotherapy
NSCLC: nonsmall-cell lung cancer
P13K: phosphatidylinositol-3-kinase
PTEN: phosphatase and tensin homolog deleted on chromosome ten
RECIST: Response Evaluation Criteria in Solid Tumors
SNP: single nucleotide polymorphisms
TNBC: triple-negative breast cancer
